# A method for addressing research gaps in HTA, developed whilst evaluating robotic-assisted surgery: a proposal

**DOI:** 10.1186/1478-4505-8-27

**Published:** 2010-09-20

**Authors:** Luciana Ballini, Silvia Minozzi, Antonella Negro, Giampiero Pirini, Roberto Grilli

**Affiliations:** 1 Agenzia Sanitaria e Sociale Regionale - Regione Emilia-Romagna (ASSR-RER), Bologna, Italy; 2 Assessorato alle Politiche per la Salute - Regione Emilia Romagna, Bologna, Italy

## Abstract

**Background:**

When evaluating health technologies with insufficient scientific evidence, only innovative potentials can be assessed. A Regional policy initiative linking the governance of health innovations to the development of clinical research has been launched by the Region of Emilia Romagna Healthcare Authority. This program, aimed at enhancing the research capacity of health organizations, encourages the development of adoption plans that combine use in clinical practice along with experimental use producing better knowledge. Following the launch of this program we developed and propose a method that, by evaluating and ranking scientific uncertainty, identifies the moment (during the stages of the technology's development) where it would be sensible to invest in research resources and capacity to further its evaluation. The method was developed and tested during a research project evaluating robotic surgery.

**Methods:**

A multidisciplinary panel carried out a 5-step evaluation process: 1) definition of the technology's evidence profile and of all relevant clinical outcomes; 2) systematic review of scientific literature and outline of the *uncertainty profile *differentiating research results into *steady, plausible, uncertain *and *unknown *results; 3) definition of the acceptable level of uncertainty for investing research resources; 4) analysis of local context; 5) identification of clinical indications with promising clinical return.

**Results:**

Outputs for each step of the evaluation process are: 1) evidence profile of the technology and systematic review; 2) uncertainty profile for each clinical indication; 3) exclusion of clinical indications not fulfilling the criteria of *maximum acceptable risk*; 4) mapping of local context; 5) recommendations for research.

Outputs of the evaluation process for robotic surgery are described in the paper.

**Conclusions:**

This method attempts to rank levels of uncertainty in order to distinguish promising from hazardous clinical use and to outline a research course of action. Decision makers wishing to tie coverage policies to the development of scientific evidence could find this method a useful aid to the governance of innovations.

## Background

Innovations pose serious challenges to Health Systems committed to delivering effective and updated health care, while resisting the pressures of unremitting modernization [1]. A significant number of technologies become available with a limited knowledge base and well before their process of evaluation is completed [2]. Decision makers have to choose between early, perhaps hazardous, adoption and delay or time-buying while further scientific evidence is being produced. Both decisions carry their own share of risk: the first decision may lead to the spread of ineffective or even harmful care, as well as unnecessary costs; the second one may cause denial of (later-proven) effective care.

The pressure to innovate and the length of time necessary for the publication of robust results make it difficult to "freeze" decisions and health organizations need criteria to discriminate really promising innovations from less advantageous ones. Moreover past experiences tell us that early adoption often proves to be a "research killer": the drive and the economic interests for the collection of further evidence diminish once the new technologies have entered clinical practice, while the ethical problems related to the recruitment and randomization of patients increase [3].

Policy options - such as Coverage with Evidence Development (CED) or Only in Research options (OIR) - adopted by Medicare in the US, NICE in the UK and other European institutions, try to combine early use in clinical practice with the development of the further research needed [3-6]. Even with these options at hand, which do not guarantee resolution of all uncertainties, the investment remains substantial and requires a balance between the risk of immature investment and the benefit of diminishing uncertainty. Tools have therefore been developed, such as the Value of Information analysis, to assess the need for further research and its potential advantages [7].

If further research can inform decisions on the adoption of new technologies, formulating the relevant and useful research questions is a crucial step for the success of the effort. Within a governmental programme, launched by our Region, the governance of innovations has been linked to the development of the research capacity of the Region's Health Trusts and Hospitals. These are encouraged, when proposing adoption of innovative technologies, to outline research projects addressing some of the unresolved questions. At present the programme does not involve coverage schemes for new technologies linked to the production of evidence, and decisions on acquisition and adoption are left to the local Health Trusts, provided they take on responsibility for prospective evaluations of the claimed clinical benefits. A substantial Regional grant has been allocated to health and clinical research and professional networks adopting new technologies are invited to submit research proposals to obtain funding.

A Regional Observatory for Innovation (ORI) has been set up by the Regional Agency for Health and Social Care (ASSR-RER) to support decision-makers and health professionals in carrying out evaluations of technological and clinical organisational innovations, both retrospectively, through systematic reviews and appraisal of evidence, and prospectively through clinical trials.

To implement this long-term research programme ORI has developed a method for the evaluation of new technologies, aimed at supporting professionals and managers in their preliminarily assessment of technologies and in formulating research proposals. This method involves assessing and ranking the scientific uncertainty surrounding a new technology and identifying the point in time, during the development of the technology, where it would be wise to invest research resources and capacity to further its evaluation.

We propose this method, piloted within a HTA project on robotic assisted surgery, which involves:

- the outline of an *uncertainty profile *of the technology, based on the systematic review of the literature,

- the development of a research programme strategy based on the knowledge needs expressed by experts and stakeholders.

The ultimate goal of this process is to advise on research initiatives necessary to fill existing knowledge gaps and not to recommend options and behaviour for routine clinical practice.

## Objective

To asses the stage of the evaluation process of an innovative health technology, identify research gaps and prioritise research questions.

### Context

Robotic-assisted surgery is a form of minimally invasive surgery carried out with a device that comprises the following components: computer console, patient-side cart and detachable instruments. The surgeon is situated at the console, several feet away from the operating table, and operates instruments attached to multiple robot arms. The robotic arms are attached to the operating table and perform the surgeon's movements translated by the computer on the patient. This robotic system provides three-dimensional visualization, intuitive movement of instruments, and 360° manoeuvrability of the tips of the instruments through the laparoscopic ports. Surgical robots are a highly expensive technology (around Euro1.8 - 2 million) and have already been introduced in two district hospitals in the Emilia-Romagna Region via donations from non-profit organizations. The initial costs were not sustained by the Regional Health System but the very high costs of use and maintenance bear down on public resources. In order to ensure safe and appropriate clinical use of the robot and to capitalize on this technology ORI undertook an evaluation [8]. Although Regional Health Trusts and Hospitals enjoy a fair degree of autonomy regarding acquisition and use of health technologies, the Regional Health Authority deemed it necessary to slow further diffusion of surgical robots and issued an official notice asking the general managers of the hospitals to restrain from acquiring more surgical robots and from accepting further donations until completion of the formal evaluation undertaken by ORI.

## Methods

The proposed method is built on a number of propositions. The first proposition is that, before starting an evaluation process, a strong theoretical case should be made for a new technology, stating the rationale for its use and detailing the *evidence profile *for the hypothetical clinical and health benefits that would make its use worthwhile. The second proposition is that the available evidence should be evaluated against this blue print of conceptual clinical effectiveness, leading to an *uncertainty profile *of the technology, which is used to highlight knowledge gaps. The third proposition is that, following the theoretical rationale for the technology and the appraisal of the evidence, a *critical *outcome or dimension is identified to determine the stage of development of the technology. The final proposition is that the research questions developed through the preceding exercise should be prioritised according to the research capacity of the local context.

### a) The theoretical rationale

Immature technologies still in the process of having their clinical place and relevance defined, are often proposed for a variety of clinical indications and the literature reports a large array of patients, diseases and outcomes [9]. It is therefore necessary to clarify the innovative elements of the technology and its potentials. Starting from the technical characteristics of the technology, a theoretical rationale for its clinical effectiveness is defined and an evidence profile is mapped to outline the research questions aimed at proving the theoretical rationale. All relevant outcomes related to technical performance, feasibility, safety, clinical effectiveness and cost-effectiveness are specified.

The decision to define relevant outcomes before analysing the scientific literature is based on the reasoning presented by the GRADE group, an international collaboration that has researched and proposed a method for the development of recommendations for clinical practice [10]. This method, discussed, critiqued and adopted by several guideline agencies, aims at a transparent process for the appraisal of evidence and the weighing up of benefits and risks of health interventions. It is claimed that discussion and agreement upon relevant outcomes, prior to reviewing the scientific literature, ensures that evaluation of the published research and subsequent development of recommendations are made explicit and unbiased by the search results. As literature on immature technologies is often limited to feasibility and safety outcomes, the initial experts' input for the definition of outcomes related to clinical effectiveness proves indispensable.

### b) The uncertainty profile

Immature technologies are commonly supported by a low quality body of evidence (i.e. case series and uncontrolled studies) unsuitable to draw any firm conclusions from. Despite this, it is still possible to explore this uncertainty. Following the traditional line of development and evaluation of a new technology - from technical performance to cost-effectiveness [11] - the Regional Observatory for Innovations developed a system for grading levels of uncertainty. The principle used to differentiate between levels of uncertainty is an adaptation of the grading of the level of evidence developed by the GRADE group. The GRADE's method for the development of recommendations for clinical practice involves assessment of both the level of evidence and of the strength of each recommendation. Quality and level of evidence is classified and graded according to whether "further research is [more or less] likely to change the level of confidence in the estimate of effect" [12]. In order to evaluate and categorize evidence drawn from low quality studies, we analysed results according to *the likelihood that further studies and of better methodological quality would change size and direction of estimates*. Using this adapted criterion, the results of the studies are classified into the following four categories:

***1. Steady results***: results that are highly unlikely to be changed by further studies

***2. Plausible results*: **consistent results coming from sufficiently numerous high quality observational studies and related to outcomes for which comparative evaluations are not strictly necessary

***3. Uncertain results*: **results that would most probably change, in both size and direction of estimate, if evaluated through randomised clinical trials

***4. Unknown results*: **unreported /non-existent results on outcomes judged by the panel to be relevant for the evaluation of the technology

Full definitions and examples for the four categories are provided in Table 1.

**Table 1:** Process of Evaluation of Immature Technology: categorisation of published research results

Level of uncertainty	Description
**Steady results **: results that are highly unlikely to be changed by further studies.	Results derived from well conducted comparative trials, i.e. systematic reviews of randomised controlled trials, several randomized controlled trials or quasi randomised trials or controlled non randomised studies with adequate adjusting for confounding factors, large sample sizes and concordant statistically significant results.
	*Example of Da Vinci robot: steady results limited to absolute costs*

**Plausible results:** consistent results, coming from sufficiently numerous observational studies, which would probably not change significantly if evaluated through randomised clinical trials.	a) consistent results derived from high quality observational studies (i.e. prospective comparative cohort studies with adequate adjusting for confounding factors) showing remarkable results for real benefits unlikely to be changed for direction of estimate by further randomised trials;
	b)consistent results related to outcomes that do not demand evaluation through comparative studies, as judgements are based on performance against absolute values or thresholds and the new technology is not required to perform better than the current alternatives.
	*Example of Da Vinci robot: plausibly stable results of a robotic-assisted surgical intervention related, for feasibility, to results showing duration of intervention consistently keeping below maximum-time duration threshold and, for safety, to results consistently showing keeping below a maximum quantity of blood-loss threshold*

**Uncertain results:** results that would most probably change, in both size and direction of estimate, if evaluated through randomised clinical trials.	Results coming from uncontrolled studies related to outcomes that need rigorous comparative studies, as their evaluation relies on differences between estimates and because the new technology is required to perform differently from the current alternatives.
	*Example of the Da Vinci robot: uncertain results related to surgical outcomes (i.e. adequate margins) and secondary clinical outcomes (such as continence, sexual potency for prostatectomy, time to nutrition for gastrointestinal surgery)*

**Unknown results**	Unreported or non-existent results on all outcomes judged by the panel to be relevant for the evaluation of the technology.
	*Example of the Da Vinci robot: unknown results related to outcomes such as disease-free time, recurrence, survival, mortality etc. not assessed by any of the published studies*

This evaluation of the evidence - similar to an "evidence mapping" exercise [13] - is carried out for each clinical use and provides an *uncertainty profile *of the technology for the different indications taken into examination

### c) Identification of the *critical *outcome/dimension

Within the research and evaluation continuum (proceeding from technical performance, feasibility, safety, clinical effectiveness to cost-effectiveness) the *critical *outcome within the *critical *domain is chosen as the boundary for judging the technology to be ready for evaluation through Phase III clinical trials. At least *plausible results *are deemed essential for this critical outcome. This imaginary line, which identifies the technology's stage of development at which it is sensible for a health service to invest research resources, sets the boundaries for the area of *acceptable maximum risk*. Dissimilar types of risk boundary would be set for different technologies, depending on their purpose, function and clinical *rationale*.

### d) Assessment of research capacity of the context

Once a list of clinical indications and relating research questions is drawn up, priority for research proposals is decided using the following criteria:

- existing high-profile professional expertise and institutes of excellence capable of employing and evaluating the technology through appropriate research programmes;

- adequate estimated size of the target population for the intended clinical use, for which a plausible beneficial clinical impact is expected.

**Figure 1:**
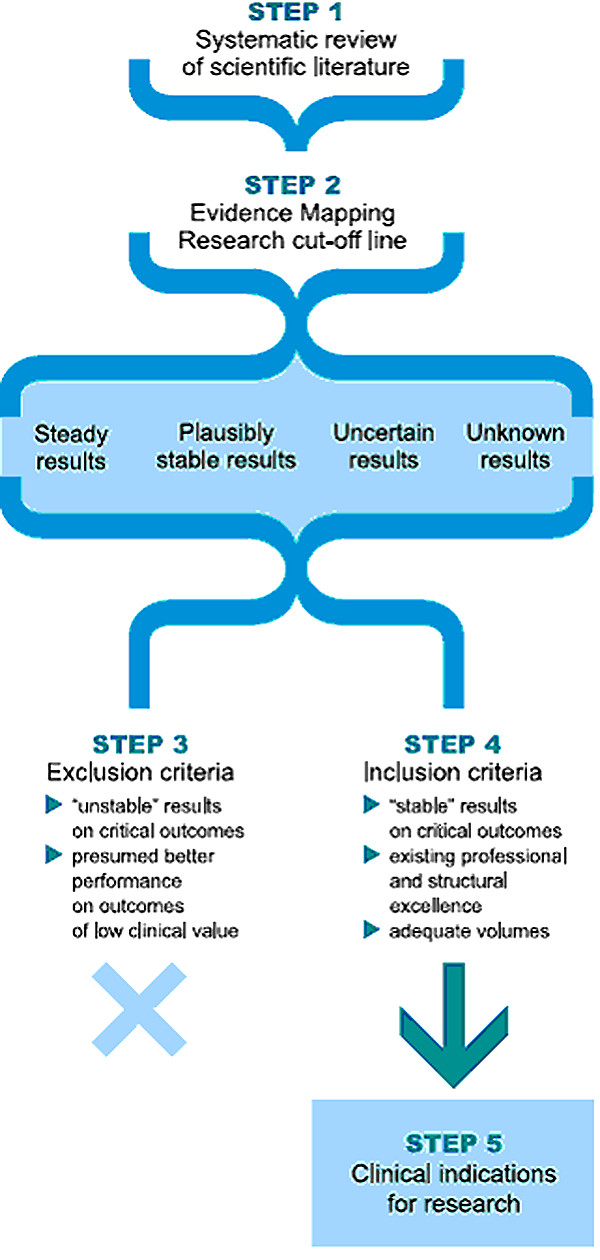
**Process for the Evaluation of Immature Technology**.

The proposed method, based on the above assumptions, is applied in a five step process (Figure 1) carried out by a multidisciplinary panel of experts. Outputs of each phase of the process used to evaluate the Da Vinci robot are described below.

## Results

### *Step 1 *- Technical description of the technology, definition of the theoretical rationale in favour of the technology and list of relevant clinical outcomes

A comprehensive description and assessment of the technical characteristics of the technology was prepared by the clinical engineers. Scope of this effort was to provide the working group with an adequate background of information to understand and appreciate the functioning of the technology, differences from existing similar technologies and advantages and improvement on technical performance. This information was also used to define the appropriate comparators.

A multidisciplinary panel of experts was convened consisting of clinicians, clinical engineers, methodologists, epidemiologists, health economists, hospital administrators and other stakeholders. Surgeons "in favour" and surgeons "against" robotic assisted surgery were invited. The panel defined the theoretical rationale in favour of the new technology as follows: robotic assisted surgery is a form of minimally invasive surgical technique that could prove more effective than others, due to its greater precision in performing demolishing and reconstructive surgical acts for interventions on very small sites, leading to presumably better surgical outcomes, less adverse effects and equivalent primary clinical outcomes.

The appropriate comparator for robotic surgery was therefore identified in other laparoscopic surgery techniques. The relevant outcomes for evaluating feasibility were chosen to be the duration of the intervention, costs, conversion to open or other laparoscopic surgery and the learning curve, while safety was assessed in terms of blood loss, need for transfusion, intra and post-operative complications. The evaluation of efficacy was decided to be based on surgical outcomes and secondary and primary clinical outcomes. In the example of the use of the Da Vinci robot for radical prostatectomy the panel defined the target population (patients diagnosed with prostate cancer in stage < = T2 eligible for radical surgery); the expected benefits (less adverse effects and equivalent clinical effectiveness); the relevant clinical outcomes (continence, sexual potency, adequate surgical margins, biochemical failure and survival).

The panel decided on the *critical *outcome/domain and set the cut-off line at the safety stage, agreeing that clinical indications lacking *plausible *results on safety would be excluded from the list of locally researchable questions

### *Step 2 - Uncertainty profile*: systematic review of the scientific literature, ranking of uncertainty and mapping of the stage of knowledge

The systematic review and appraisal of published studies was developed in two phases. An initial systematic search and appraisal of all published HTA reports, guidelines, systematic reviews and meta-analysis was carried out to obtain a good overview of all evaluated clinical uses. The initial systematic review of the literature review on robotic surgery produced a fairly long list of surgical interventions in which robots had been tested, ranging from very simple (cholecystectomy) to more complex ones (cardio-thoracic surgery, paediatric surgery).

The results of the review were presented to the panel and members were asked to identify the clinical indications of greatest interest considering the following: the clinical *rationale *of the technology, the volume and quality of existing literature for each clinical indication and the context capacity to "accommodate" the technology's clinical use. On the basis of these the panel decided that the second phase of systematic reviews of all primary studies should be carried out on the use of the Da Vinci robot in urological, abdominal, gynaecological, bariatric and thoracic surgery. The specificity of the local context thus did impact on the subsequent research questions and content of the report as indications such as heart surgery were excluded, due to the absence of local expertise in minimally invasive surgery in this field. This is justified by the fact that any future investment must be linked to and based on the real potentials of use offered by the context hosting the new technology. A further systematic review of all published primary studies was then carried out on this subsection of clinical indications. All retrieved studies were assessed and synthesised in tables of evidence reporting study design, number of patients, comparators and estimates for outcomes.

**Figure 2:**
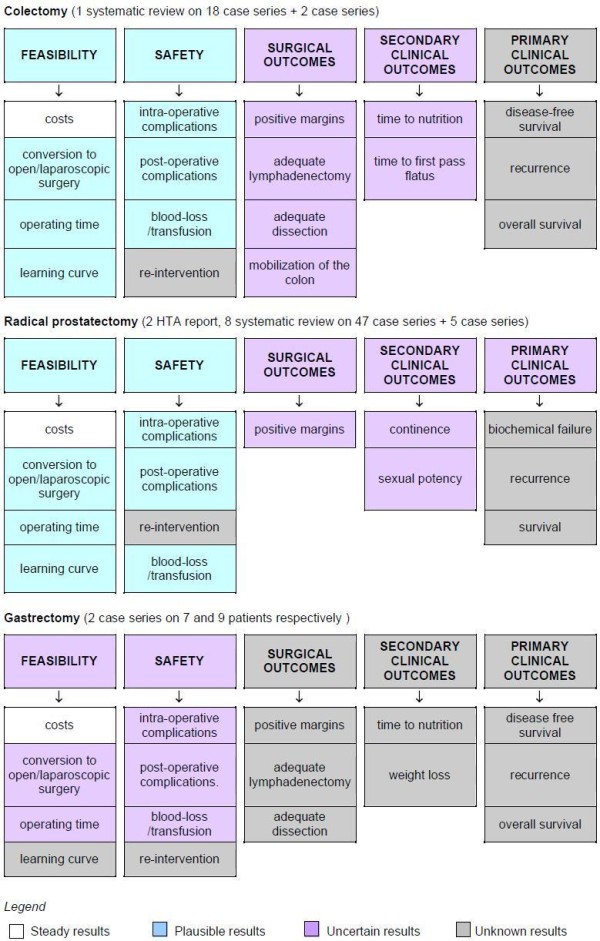
**Uncertainty Mapping: example of the Da Vinci robot**.

Results of the studies were then graded according to the different levels of uncertainty, providing an *uncertainty profile *of the technology for each clinical indication taken into examination (see example in Figure 2).

The value of this exercise was manifold:

- It allowed the stage of development of the technology to be charted on the research continuum and to compare stages reached by different clinical indications.

- It provided a blue print for future research questions.

- It provided the parameters for future updating of the literature review.

### *Step 3*: Exclusion of clinical indications

On the basis of the *uncertainty profile *the panel decided which clinical indications of the technology were to be excluded from the evaluation process. Two types of exclusion criteria were applied.

The first one was determined by the cut-off line for the *acceptable maximum risk*, set by the panel at the safety dimension, which consented the exclusion of the clinical indications with no *plausible results *on safety outcomes (blood loss, intra-operative complications, conversion to open surgery etc.). The concept of *plausibility *helped to deal with uncertainty and discriminate potentially promising from hazardous use of a technology. (i.e. gastrectomy, lobectomy, esophagectomy etc.).

The second criterion for exclusion was based on the importance attributed to the clinical outcomes: indications were excluded when the presumed better performance of the technology was related to clinical outcomes judged by the panel to be of limited importance. Further clinical indications were thus excluded by the panel (for example cholecystectomy) as the presumed advantages over laparoscopic surgery were considered of insufficient clinical value.

While the exclusion based on the lack of *plausible results *is guided by the analysis of the literature, the exclusion based on the importance of the outcomes implies a value judgement expressed by the panel at the outset of the process.

Step 3 concluded with a list of clinical indications for which the technology was considered ready to be evaluated through comparative effectiveness trials (radical prostatectomy, colectomy, hysterectomy, fundoplication, bariatric surgery, thymectomy). The remaining indications were excluded because in need of further trials evaluating safety or because suggesting minor clinical benefit.

### *Step 4*: prioritisation of clinical indications relevant for the local context

The range of clinical indications was further narrowed down using characteristics of context as criteria for the prioritisation of relevant clinical questions.

Output of step four was a context mapping aimed at evaluating the presumed clinical impact of the technology on the local health system. Regional data reports on patients undergoing the different surgical procedures, for which robotic surgery showed important potentials, were produced, as well as reports on volumes of activity and performance of all hospitals' surgical units. This helped to identify the centres that could be suitable candidates for experimental use of this technology. On the basis of this analysis further indications were excluded as they did not satisfy the criterion of feasibility for clinical trials, because of small size of target population or the lack of existing high-profile professional expertise and institutes of excellence capable of employing and evaluating the technology through appropriate research programmes.

### *Step 5*: Recommendations for clinical research

Coherently with the limited knowledge that accompanies immature technologies, assessment reports cannot come to firm conclusions on the opportunity to adopt a technology or not. They can nevertheless provide information supporting restrain of unjustified diffusion in clinical practice, while endorsing clinical use within an experimental setting. Output of step five was the list of clinical indications appropriate for Regional research programmes. At the end of the process, the proposed areas of research for the experimental use of the Da Vinci robot in our Region were decided to be radical prostatectomy, colo-rectal surgery and bariatric surgery. A summary of the process and outputs for each one of the 5 steps is given in Table 2.

**Table 2:** Process of Evaluation of Immature Technology: output of the 5 steps

Step	Process	Output
Step 1	- Technical description of the technology	- Evidence profile
	- Definition of relevant outcomes and grading of studies	
	- Identification of "critical outcome" setting the boundary for the acceptable maximum risk	

Step 2	- Systematic review of literature	- *Uncertainty profile*
	- Analysis of quantity and quality of published research results by clinical indication for each outcome	

Step 3	- Application of exclusion criteria:	- List of excluded clinical applications
	a) cut off-line above which level of uncertainty is considered too high to carry out research programmes;	
	b) outcomes considered of insufficient clinical value	

Step 4	- Production of reports on:	*- Context mapping*
	a) data on volumes of activity and size of potential population target	
	b) distribution of organisational excellence and professional expertise	

Step 5	- Selection of clinical indications with promising clinical returns and reflecting local expertise and activity	- Recommendations for research

## Discussion and Conclusions

New health technologies and innovations in general pose the problem of governance and every health service or organization has experienced the disappointment of having innovations introduced and diffused without being able to rely on sufficient information. The problem is particularly felt with immature technologies which invariably elicit enthusiasm, competition and desire to pioneer.

The unplanned spread of use of technologies makes decisions on coverage compelling before formal processes of approvals have been completed. In these situations withdrawal or cessation of services might prove difficult, as well as unpopular, and tying adoption policies to the development and acquisition of scientific evidence on the technology's clinical impact might be a good way to manage momentum.

During a research project involving the evaluation of a costly and emerging health technology (the surgical Da Vinci robot) we developed and piloted a method that specifically dealt with health innovations equipped with an incomplete body of empirical knowledge. The main assumption of the described method is that the introduction of emerging health technologies into health services and organisations ought to be related to long term strategies of research and development and to the capacity of the services and organizations to carry out clinical and health research [14]. The proposed method is thus a formal process that defines experimental use of a technology within a health system and that, by putting the technology on "probation" for a given time, tries to control its unplanned spread and use.

While the Regional Health Authority issued an official notice that stopped further acquisitions of Da Vinci robots, no reimbursement restrictions were made for the use of the existing ones. One of the objectives of the evaluation was therefore to agree on the clinical indications for which the robot should *not *be used and, as a first result, these robotic-assisted surgical interventions, like cholicystectomy, were suspended by hospitals and surgeons.

The recommendations for research, final output of the five-step process, did not involve an economic evaluation. Our method cannot compete with the more sophisticated approaches that put the cost of research in relation to the value of the benefits gained should the technology prove effective [15,16]. Cost-effectiveness and Value of Perfect Information analyses were beyond the scope of the mandate as a comparison between investments on different technologies was not required [17].

A much less ambitious objective was to agree on a list of clinical indications for which a case for promising clinical returns could be put forward and for the evaluation of which the local context should take responsibility.

Off-shoot of this work has been the submission of a proposal, by a group of local surgeons, for two multicentre clinical trials aimed at comparing clinical effectiveness of robotic surgery versus laparoscopic surgery in patients treated surgically for colo-rectal cancer and prostate cancer.

### Strengths

We believe that the strength of the method we are proposing lies in its relying on scientific literature, no matter how scanty and inadequate. We took this as a starting point to assess what has been studied so far, what needs to be researched and the most appropriate time for the Health Service to take direct responsibility for the *further research needed*. In this approach uncertainty is not taken to be a homogeneous state of affairs, but is ranked according to varying levels in order to define and choose the tolerable level of uncertainty for engaging in the furthering of knowledge.

An additional strength of this method we believe to be the role of the experts. When developing clinical practice guidelines experts' opinions are combined with scientific evidence and used to grade certainty or *confidence *that "the desirable effects of intervention will outweigh the undesirable effects" [18]. When evaluating emerging technologies panel members are asked to grade *uncertainty *and state how close or far current research is to answering their own most relevant questions. The outcome of this appraisal is obviously not to recommend a clinical course of action, but to chart a research course of action.

### Limitations

In developing this methodology we did not address the issue of prioritisation of assessment and did not attempt to develop criteria to decide whether or not an emerging technology is worthy of such an evaluation process. Given the quantity of stimuli and proposals for innovations competing for attention, a prioritisation method needs to be developed, possibly based on preliminary background information and on criteria suitable for scaling probability of uncontrolled spread.

Our report contained only rudimental analysis of costs. As no cost-benefit, cost-opportunity or cost-effectiveness analyses were carried out, the method used did not address economic evaluation of possible research returns. Nevertheless the research areas of top clinical priority could be further evaluated and ranked in this way.

Limits of generalisability of the outcome of this process could be given by the panels' definition of the important outcomes and the cut-off line for the acceptable risk, but we believe this limit is mitigated by the explicitness of the process and the clinical *rationale *on which it is based.

Limits related to the weight of the characteristics of the context are on the other hand inherent in the specificity of policy decisions and the method aims at setting common criteria for selecting relevant issues of the context.

## Competing interests

The project was partly funded by a research grant from the Italian Ministry of Health (Programma "Ricerca Sanitaria Finalizzata ex art.12 DLgs 502/92 - grant N° RFPS - 2007 -8-640752) and by the ASSR-RER. LB, SM, AN and RG are employed by the ASSR-RER. However the views expressed are those of the authors and not necessarily those of the funding bodies and all authors had no personal interests in the results of this project.

## Authors' contributions

LB conceptualized the study, developed the method, conducted the analysis for the ranking of uncertainty, wrote the first, subsequent and final drafts of the manuscript; SM conducted the systematic review and critical appraisal of the literature and made substantial contribution to the drafts of the manuscript; AN performed the data analysis; GP conducted the review and assessment of the relevant technical literature; RG made substantial contribution to the conception of the study and methodology and to the analysis of outputs. All authors have read and approved the manuscript.
